# Improving motor neuron-like cell differentiation of hEnSCs by the combination of epothilone B loaded PCL microspheres in optimized 3D collagen hydrogel

**DOI:** 10.1038/s41598-021-01071-2

**Published:** 2021-11-05

**Authors:** Narges Mahmoodi, Jafar Ai, Zahra Hassannejad, Somayeh Ebrahimi-Barough, Elham Hasanzadeh, Houra Nekounam, Alexander R. Vaccaro, Vafa Rahimi-Movaghar

**Affiliations:** 1 Sina Trauma and Surgery Research Center, Tehran University of Medical Sciences, Tehran, Iran; 2 Department of Tissue Engineering and Applied Cell Sciences, School of Advanced Technologies in Medicine, Tehran University of Medical Sciences, Tehran, Iran; 3 Pediatric Urology and Regenerative Medicine Research Center, Tissue, Cell and Gene Research Institute, Tehran University of Medical Sciences, Tehran, Iran; 4 Immunogenetics Research Center, Department of Tissue Engineering and Applied Cell Sciences, School of Advanced Technologies in Medicine, Mazandaran University of Medical Sciences, Sari, Iran; 5 Department of Medical Nanotechnology, School of Advanced Technologies in Medicine, Tehran University of Medical Sciences, Tehran, Iran; 6 Department of Orthopedic Surgery, Rothman Institute, Thomas Jefferson University, Philadelphia, PA USA

**Keywords:** Cell biology, Developmental biology, Neuroscience, Stem cells, Neurology

## Abstract

Spinal cord regeneration is limited due to various obstacles and complex pathophysiological events after injury. Combination therapy is one approach that recently garnered attention for spinal cord injury (SCI) recovery. A composite of three-dimensional (3D) collagen hydrogel containing epothilone B (EpoB)-loaded polycaprolactone (PCL) microspheres (2.5 ng/mg, 10 ng/mg, and 40 ng/mg EpoB/PCL) were fabricated and optimized to improve motor neuron (MN) differentiation efficacy of human endometrial stem cells (hEnSCs). The microspheres were characterized using liquid chromatography-mass/mass spectrometry (LC-mas/mas) to assess the drug release and scanning electron microscope (SEM) for morphological assessment. hEnSCs were isolated, then characterized by flow cytometry, and seeded on the optimized 3D composite. Based on cell morphology and proliferation, cross-linked collagen hydrogels with and without 2.5 ng/mg EpoB loaded PCL microspheres were selected as the optimized formulations to compare the effect of EpoB release on MN differentiation. After differentiation, the expression of MN markers was estimated by real-time PCR and immunofluorescence (IF). The collagen hydrogel containing the EpoB group had the highest HB9 and ISL-1 expression and the longest neurite elongation. Providing a 3D permissive environment with EpoB, significantly improves MN-like cell differentiation and maturation of hEnSCs and is a promising approach to replace lost neurons after SCI.

## Introduction

Spinal cord injury (SCI) is a very common traumatic event and is mainly caused by falls and motor vehicle accidents (MVAs)^1,2^. Extensive neuron loss and axon degeneration are common cellular findings at the lesion site following SCI, leading to severe disability in patients due to limited neuronal regeneration and recovery^3–5^.

Currently, no therapy is available for complete functional recovery after SCI^6,7^, however, advances in medical care can increase life expectancy in patients with SCI and reduce their families' financial burden^8,9^. As a growing research area, using various stem cells has been garnered great attention for nerve tissue regeneration through cell differentiation for lost cell replacement, anti-inflammatory effects, and neurotrophic factor secretion^4,10^.

However, stem cell therapy utility is usually limited given low cell survival and uncontrollable cell differentiation at the site of injury^4^. SCI also leads to complex, destructive biological processes that may continue up to one year after injury^11^. Thus, to reach the full potential of stem cells, a synergistic effect through combination therapy is necessary^12^.

One of the most promising stem cells for SCI therapy is a group of adult multipotent mesenchymal stem cells (MSCs), which can be induced to different cell types^13–15^. Human endometrial stem cells (hEnSCs) as a MSCs have considered attention for cell therapy and tissue engineering^16–20^. Interestingly, in a recent study, a comparison between hEnSCs and human bone marrow stem cells (hBM-MSCs) showed a better motor neuron (MN) differentiation potential for hEnSCs^21^. Improvement in differentiating stem cells into specialized neurons is one solution for replacing damaged or lost cells, thereby leading to potential functional recovery^22^.

Motor neurons are a type of nerve cells that end at myocytes and transfer nerve impulses to control voluntary muscle movements. After SCI, there is a substantial loss of motor neurons and interneurons in the spinal cord tissue^23^, while endogenous regeneration of neurons and revival of motor function is limited^24^. A spectrum of illnesses such as spinal muscular atrophy (SMA) and amyotrophic lateral sclerosis (ALS) can also lead to motor neuron degeneration^25^. To cure these illnesses, researchers are trying to develop methods to produce MN-like cells from stem cells^26^. MN-derived cells can also be utilized for MN-modelling and drug-screening assays^27,28^.

Microtubules (MTs) are a type of cytoskeletons that, as a structural component, are essential for cellular polarization, neuronal morphology, axonal regeneration, motility, protein secretion, and intracellular movement^29,30^. MTs disruption and disorganization are characteristics of damaged nerve cells after SCI^31,32^. Likewise, MTs disorganization has been noticed as a primary etiology of neurodegenerative diseases such as Alzheimer's and Parkinson's diseases^33,34^, and, recently, microtubule-stabilizing agents (MSAs) have been considered in the treatment strategy of these disorders^35^.

Epothilone B (EpoB), also known as patupilone, is a macrolide derived from myxobacteria *Sorangium cellulosum* and an MSA that can cross the blood–brain barrier (BBB). A recent study showed that EpoB can induce axon regeneration and elongation in damaged nerve cells and decrease scar formation after SCI in rodents^36–38^. These studies, however, used systemic administration of drugs, and the possible side effects and short half-life limit us from reaching maximal efficacy. Moreover, various injections require invasive medical intervention. A biocompatible and biodegradable delivery system can offer sustained release of therapeutic agents and thus increase their effectiveness without using excessive invasive surgical techniques^24^. For the delivery of drugs, they can be encapsulated into biomaterial-based microspheres for sustained release, which provides in situ sustained release^39^.

In the field of tissue engineering, utilizing naturally derived hydrogels have gained momentum for localized drug delivery application for presenting easily mimicking and integrating with native tissues, for similar characteristics to soft tissues, and their excellent recognition by different cell types^40–42^.

Among various natural-based hydrogels, collagen is the major protein of the extracellular matrix (ECM) in many mammalian tissues^43^. Collagen is distinguished being a highly biocompatible cellular growth supporter and is noncytotoxic^44^. Though collagen has been extensively used in the clinic and tissue engineering for various applications^45^, its high degradation degree, mechanical instability, and thermosensitivity have led researchers to add other polymers or cross-linkers to achieve more desirable features^40,46^. Therefore, in this study, we chose a cross-linking approach to improve hydrogel stability while preserving the utility of collagen hydrogel as an appropriate three-dimensional (3D) structure for enhancing cell proliferation and differentiation. Many different kinds of cross-linked hydrogels have been used as 3D scaffolds and drug delivery systems for soft tissue engineering, including corneal, cartilage, skin, tendon, and peripheral and central nervous tissues^47–49^.

Proanthocyanidin (PA) is a natural polyphenolic oligomeric flavonoid found in flowers, vegetables, fruits, seeds, nuts, and barks^50,51^. PA is a promising cross-linker with a variety of biological activities, including anti-inflammatory, anti-tumor, antioxidant, anti-bacterial, anti-calcification. PA provides biocompatible cross-linked hydrogels and has non-toxicity properties, unlike common cross-linkers such as formaldehyde and glutaraldehyde^52–55^.

Evidence shows that combination strategies achieve greater results than using each component separately by addressing various aspects of SCI pathophysiology^12^. Accordingly, in this study, we aimed to enhance the differentiation potential of hEnSCs into MN-like cells and axonal elongation using the signaling molecules sonic hedgehog (SHH) and retinoic acid (RA), and also by providing a hydrogel-based 3D microenvironment capable of controlling the release of EpoB using PCL microspheres.

## Materials and methods

### Isolation and culture of human endometrial stem cell (hEnSC)

hEnSCs were isolated according to a previous study^56^. The collection of discarded endometrium biopsies from patients was achieved via the informed consent from the patients/legal guardian. All methods were performed in accordance with the relevant guidelines and regulations of Tehran University of Medical Sciences and approved by the university's ethical committee (code: IR.TUMS.REC.1394.1137). Briefly, biopsies of endometrium were washed with phosphate-buffered saline (PBS; Sigma, P4417) containing 2–3% amphotericin B and penicillin/streptomycin, were cut into small pieces and digested with collagenase type I (1 mg/mL, Sigma-Aldrich) in Dulbecco's Modified Eagle Medium/Nutrient Mixture F-12 (DMEM-F12; Invitrogen) for 2 h at 37 °C. The enzyme was then neutralized by adding a complete medium composed of DMEM-F12 with 10% fetal bovine serum (FBS; Gibco, 10270-106). Subsequently, the samples were passed through 70 μm and 40 μm filters and were centrifuged at 1200 rpm for 5 min. After the supernatant was removed, the pellet of cells was resuspended in a complete medium containing 10% FBS and 1% penicillin/streptomycin. The cell suspension was transferred to 6-well cell culture plates and incubated in a humidified chamber at 37 °C with 5% CO_2_. The medium was exchanged every 3 days. Three or four passages of hEnSCs were used for all of our experiments.

### Flow cytometry

To confirm the identity of the isolated cells, passage three cells were used for flow cytometry BD FACSCalibur (BD Biosciences, USA). The evaluated markers were as follows: endometrial stem cell marker (CD146), mesenchymal stem cell markers (CD44, CD90, and CD105), hematopoietic stem cell markers (CD34 and CD45), and endothelial stem cell markers (CD31).

The cells were washed three times with Hank's Balanced Salt Solution (HBSS) and 2% bovine serum albumin (BSA; Sigma, A7030) and then were incubated with a specific antibody (Table 1) at concentrations recommended by the respective manufacturers (for 1 h). Data were evaluated by FlowJo Version 7 software.

**Table 1:** Markers for hEnSCs identification.

	Cell surface marker	Percentage (%)	Antibody	Catalog number
Endometrial stem cell marker	CD146	89.1	PE-conjugated anti-CD146	BD Biosciences, 561013
Mesenchymal stem cell marker	CD105	98.4	PerCP-conjugated anti-CD105	BioLegend, 323216
	CD90	98.4	FITC-conjugated anti-CD90	Exbio, 1F-652-100T
	CD44	98.5	FITC- conjugated anti-CD44	Immunostep, 44F2-100T
Hemopoietic stem cell marker	CD45	0.3	FITC-conjugated anti-CD45	BD Biosciences, 560976
	CD34	0.8	PE-conjugated anti-CD34	Exbio, 1P-664-T025
Endothelial marker	CD31	0.3	PE-conjugated anti-CD31	Immunostep, 31PE-100T

*PE* phycoerythrin, *PerCP* peridinin chlorophyll protein, *FITC* fluorescein isothiocyanate.

### Green fluorescent protein (GFP) labeled hEnSCs

Based on previous studies, lentivirus (second-generation) was used for GFP gene transduction of hEnSCs and HEK293-T cells^57^. We also labeled HEK293-T as the standard cell line with high efficiency for GFP labeling. The pCDHCop GFP-Pru plasmid was used for labeling, which contained EF-1α promotor to upregulate the expression of GFP transgene. For transient transfection of HEK293-T, the vector associated with psPAX2 as a packaging plasmid and pMD2.G as an envelope plasmid were used via the calcium phosphate precipitation method. Then, the viral particles were harvested from the supernatant for 3 days, then stored at − 80 °C. HEK293-T (30% confluency) and hEnSCs (50% confluency) were then transduced by adding 5 μg/mL polybrene and lentiviral particles. Labeled cells were selected using 10 μg/mL puromycin, which was added to DMEM-F12. GFP expression was observed in hEnSCs via fluorescent microscopy after 3 days.

### Microsphere fabrication and sterilization

The oil in water (o/w) single emulsion method was used to synthesize the EpoB-loaded PCL microspheres. First, 250 mg of polycaprolactone (PCL; Sigma, 363170) was dissolved in 2 mL of dichloromethane (DCM; Sigma, 32222) using a magnet stirrer (Heidolph, Hei-Tec) for 25 min at 35 °C. Three groups of EpoB-loaded PCL microspheres were fabricated by adding 0.625 µg, 2.5 µg, and 10 µg of EpoB (Abcam, 152044-54-7) to the PCL solution for an EpoB loading of 2.5 ng/mg, 10 ng/mg, and 40 ng/mg (EpoB/PCL), respectively. Unloaded microspheres were synthesized through the elimination of EpoB from the formulation. 2 mL of pure ethanol (Merck, 111727) was added to the solution while stirring, and 2 mL of 2% (w/v) polyvinyl alcohol (PVA) (Sigma, 363170) solution was slowly added drop by drop without disrupting the boundary layer. Then, the solution was mixed and emulsified for 12 s using a vortexer (Heidolph) and instantly added to a 100 mL beaker containing 50 mL of 0.3% (w/v) PVA solution at 35 °C. To evaporate the organic solvent, the solution was stirred with a 3 cm × 0.5 cm round magnetic stirring bar at 500 revolutions per minute (rpm) for 4 h in a 100 mL beaker. Since EpoB is light sensitive, the entire fabrication process was performed in a dark room. It is worth noting, due to the importance of mentioning parameters such as magnetic bar’s length, diameter, and the size of cylindrical container for having a reproducible particle synthesis method, these specifications were mentioned above^58^.

The synthesized microspheres were collected through centrifugation at 4000 rpm (Eppendorf, 5810R) for 10 min and were washed four times with distilled water and centrifugation at 4000 rpm for 5 min each time to remove residual PVA. Then, the microspheres were frozen at − 80 °C, lyophilized for 24–48 h, and were stored at − 20 °C. For cell culture experiments, microspheres were sterilized by air-plasma for 30 s on low power (Harrick, PDC-32G).

### Microsphere characterization

#### Scanning electron microscopy (SEM)

To evaluate the size and morphology of microspheres, a ZEISS DSM 960A Oberkochen (Germany) scanning electron microscope (SEM) was used. Microspheres were sputter-coated with gold (20 kV for 4 min), and after imaging, their diameters were quantified using ImageJ software. The distribution of microsphere diameters was plotted as a histogram. Each sample was displayed from smallest to largest diameter.

#### Determination of encapsulation efficiency (EE)

After synthesizing 2.5 ng/mg, 10 ng/mg, and 40 ng/mg (w/w, EpoB/PCL) EpoB microspheres, the suspension of each group was centrifuged at 4000 rpm for 10 min at room temperature to separate the microparticles and supernatant. The amount of EpoB in the supernatant was evaluated as the extent of unencapsulated EpoB using liquid chromatography-mass/mass spectrometry (LC-Mas/Mas; HPLC Alliance 269S Waters, Quattro Micro API micromass micromass) with a C18 column (4.8 × 150 mm, 5 µm, Agilent Zorbax XDB). The standard solutions of EpoB and each microsphere sample were loaded to LC–MS/MS and first eluted with methanol: water (40:60) for 5 min at the speed of 0.5 mL/min and then methanol:water (95:5). The retention time of EpoB was 9 min. The samples were quantified based on the standard curve of EpoB (all samples were run in triplicate).

The encapsulation efficiency (EE) of EpoB in the PCL microparticle was determined as the following formula:

$${\text{EE}}\% = \frac{Total \;Epo \;B\; added\;(ng) - Unbound \;Epo\; B \;(ng)}{{Total\; EpoB\; used\; (ng)}} \times 100\%$$

#### EpoB release measurement

10 mg of 2.5 ng/mg, 10 ng/mg, and 40 ng/mg (w/w, EpoB/PCL) EpoB-loaded microspheres were suspended in 1.5 mL of PBS in a Costar^®^ Spin-X^®^ centrifuge tube filter (0.22 µm pore CA membrane; Corning, 8160). The microspheres were placed in the above chamber containing the filter, filled with 0.5 mL PBS, and the tube filled with 1 mL PBS. These tubes were used to prevent microspheres from being mistakenly collected at the time of sampling. The Costar^®^ Spin-X^®^ centrifuge tubes were then placed on a shaker (Lab Tech, LSI-3016R) at 100 rpm and incubated at 37 °C. For the in vitro study, to simulate the conditions of exchanging the culture medium, in the release test, the PBS was exchanged every 2 days. Three samples were considered for each time point (7-time points: 1 and 18 h and 1, 3, 6, 14, and 21 days). The PBS of each sample was collected entirely at each time point. First, the PBS of the tubes was collected (not the above chamber that contained the filter), then each chamber was centrifuged with its tube at 4000 rpm for 5 min until the PBS in the chamber inter the tube and could be collected. The PBS was then stored at − 80 °C and protected from light until all the samples were collected for further analysis.

The amount of EpoB was determined by using LC-Mas/Mas. In addition, to better identify the drug amount, the samples were first concentrated six times. For this purpose, each 1.5 mL samples were freeze-dried for 24–48 h and then dissolved in 200 µL deionized water and 50 µL methanol. For cell culture application, the candidate microspheres were then sterilized by air plasma (Harrick, PDC-32G) on low power for 30 s.

### Determining the effect of microspheres on cell viability and proliferation

According to experimental release results, the microsphere containing 2.5 ng/mg (EpoB/PCL) was selected as the candidate microsphere for this study, referred to as Mic-2.5-EpoB in this text. To determine the biocompatible concentration of microparticles for hEnSCs culture, first, different concentrations of unloaded PCL microspheres (5, 10, 15, and 40 mg/mL) were used by indirect MTT assay for 24, 48, and 72 h. After evaluating their results, a second indirect MTT assay was performed for Mic-2.5-EpoB for 24, 48, and 72 h to assess the effect of the loaded microsphere on viability and proliferation of hEnScs. For both MTT assays, 2 × 10^5^ hEnSCs were seeded in each 24-well cell culture plate containing 500 µL of complete medium and incubated at 37 °C, 5% CO_2_ for 24 h. Then, the cell culture was replaced with extract solutions of the microspheres and incubated at 37 °C, 5% CO_2_ for 24, 48, and 72 h. The microsphere extract solutions of the (5, 10, 15, and 40 mg/mL unloaded PCL microspheres and Mic-2.5-EpoB) in complete medium (DMEM-F12 + 10% FBS) were collected every 24 h (each group was performed in triplicate). For each time point, the medium was removed and replaced with 5 mg/mL tetrazolium (Sigma) in PBS (MTT solution) and incubated for 3 h at 37 °C, 5% CO_2_. The optical density (OD) of formazan dye dissolved in dimethyl sulfoxide solution (DMSO) was obtained using an ELISA microplate reader (Gen5, Power Wave XS2, BioTek, USA) at 590 nm. The following formula calculated the cell viability percent of hEnSCs:

$$\% {\text{Cell }}\;{\text{Viability}} = \frac{{{\text{OD }}\;{\text{treated }}\;{\text{well}}}}{{{\text{OD}}\;{\text{ control }}\;{\text{well}}}} \times 100$$

The MTT test was also used to determine the permissible particle limit in the cell culture for hEnSCs.

### Fabrication of collagen hydrogels

Collagen type I was extracted from rat tails following a previously developed method^59^. Collagen was sterilized through dialysis with 1% chloroform (Merck) using dialysis bags with a molecular cut of 6–8 kDa (SpectraPor, 132660) for 1 h at 4 °C.

Collagen hydrogels were fabricated using a ratio of 8:1:1 of sterile collagen solution:10 × DMEM/F12:HSS buffer. The HSS buffer was prepared by dissolving 4.77 g HEPES and 2.2 g sodium bicarbonate in 100 mL of 0.5 M sodium hydroxide. For gelation, 500 µL of the prepared mixture was poured into 24-well cell culture plates and incubated at 37 °C for 1 h. Two final concentrations of collagen were used to fabricate the hydrogels: 2 mg/mL and 4 mg/mL.

To improve stability of the hydrogel, collagen hydrogels were also cross-linked using different concentrations of proanthocyanidin (Grape Seed, Proanthocyanidins, Shari^®^, Iran) in PBS, including 0.25% PA, 0.5% PA, and also 0.5% PA with 0.05 M Ca(OH)_2_ (PA-Ca(OH)_2_). In the last formulation, we used Ca(OH)_2_ based on evidence indicating that Ca(OH)_2_ facilitates the penetration of PA and collagen cross-linking^52^. After one-hour incubation of collagen solutions (2 mg/mL and 4 mg/mL) at 37 °C, 100 µL of PA solution was added, and the cell culture plates were further incubated for 48 h at room temperature in a sterile condition. Then, the hydrogels were washed with PBS three times to remove the excess crosslinkers. The specifications of hydrogel preparation are summarized by code in Table 2.

**Table 2:** The code used for different hydrogels prepared through thermal and chemical cross-linking.

Hydrogel code	Desorption
Collagen	Collagen hydrogel
2-Col/PA-CH	Cross-linked 2 mg/collagen hydrogel with 0.5% proanthocyanidin and calcium hydroxide
4-Col/PA-CH	Cross-linked 4 mg/collagen hydrogel with 0.5% proanthocyanidin and calcium hydroxide
2-Col/PA-CH/Mic EpoB	Cross-linked 2 mg/mL collagen hydrogel with 0.5% proanthocyanidin (PA) and calcium hydroxide and 2.5 ng/mL Epo B microspheres
4-Col/PA-CH/Mic EpoB	Cross-linked 4 mg/mLcollagen hydrogel with 0.5% proanthocyanidin (PA) and calcium hydroxide and 2.5 ng/mL Epo B microspheres

### Fabrication of collagen hydrogel containing EpoB-loaded microspheres

To mix EpoB microspheres with collagen hydrogel for 3D culture application, candidate microspheres were coated with a dilute sterilized collagen solution (0.5 mg/mL) overnight at 4 °C. Before coating, the collagen solution was neutralized using a 1 N solution of filter-sterilized NaOH in water (syringe-filter 0.22-µm membrane), bringing the pH to around 7.4.

### Cell seeding within the collagen hydrogels

In each 24-well culture plate, 50 µL of 1 × 10^5^ hEnSCs in complete medium (DMEM-F12 and 10% FBS) was slowly added to collagen hydrogels and incubated for 30 min at 37 °C for cells attachment. Then, 150 µL complete medium was added to each well.

#### Cell morphology, attachment, and proliferation on the collagen hydrogels

##### Fluorescent microscopy

To study cell adhesion, morphology, and proliferation in collagen hydrogels, two types of GFP-labeled cells (HEK293-T and hEnSCs) were used. In each 24-well cell culture plate, 1 × 10^5^ cells were seeded and incubated at 37 °C with 5% CO_2_. To observe the cells, an Olympus (BX51, Japan) immunofluorescence microscope was used.

##### Scanning electron microscope

An SEM (AIS2300C SEI, Korea) at 20 kV was used for investigating cell attachment of hEnSCs on collagen, 2-Col/PA-CH, and 2-Col/PA-CH/Mic EpoB hydrogel scaffolds, after 48 h of cell seeding. The SEM was also performed for observing the surface morphology of collagen, 2-Col/PA, 2-Col/PA-CH, and 2-Col/PA-CH/Mic EpoB hydrogel scaffolds. The medium of each 24-well was discarded, and each specimen was washed with PBS and fixed with Karnovsky's fixative solution (consisting of 2% paraformaldehyde and 2.5% glutaraldehyde) at room temperature for 1 h. Subsequently, the prepared samples were washed twice with PBS and then dehydrated with a graded ethanol series of 30, 50, 70, 90, 95, and 100%. Afterward, to examine the specimens via SEM, the samples for cell attachment study were first dried on an aluminum foil at room temperature, and the samples for surface morphology study were freeze-dried for 48 h, then each sample was under vacuum and finally coated with gold sputtering.

### Hydrogel biodegradation degree

We assessed the degradation degree of collagen, 2-Col/PA-CH, and 2-Col/PA-CH/Mic EpoB hydrogels based on their weight loss in DMEM-F12 medium at 37 °C. The DMEM-F12 medium was exchanged every 3 days. Hydrogel weight alterations were evaluated over 14 days (all time points were run in triplicate). The degradation degree was calculated using the following equation:

$${\text{Degradation}}\;{\text{degree }} = \frac{{(W_{0} - W_{t} )}}{{W_{0} }} 100\%$$

where, *W*_*0*_ and *W*_*t*_ indicate the weight of hydrogels before and after immersing in medium, respectively.

### Swelling degree

The swelling degree of the hydrogel scaffolds, including collagen, 2-Col/PA-CH, and 2-Col/PA-CH/Mic EpoB were measured for 96 h. The hydrogels were allowed to swell in DMEM-F12 at 37 °C. Each time point was performed in triplicate.

The weight of the wet samples was measured, and the swelling degree was obtained via the following formula:

$${\text{Swelling}}\;{\text{degree}} = \frac{{(W_{t} - W_{0} )}}{{W_{0} }} \times 100$$

where, *W*_*0*_ and *W*_*t*_ are the weight of the primitive hydrogel and the weight of the swollen hydrogel, respectively.

### ATR-FTIR spectroscopy

Proanthocyanidin (PA; GrapeSeed, Shari^®^, Iran) blended tablets were first dissolved in water. Then the precipitated additives of the tablet were removed and filtered through a 0.22 μm membrane. The samples (PA, Collagen, 2-Col/PA, and 2-Col/PA-CH) were freeze-dried for 48 h and then analyzed using an ATR accessory (Nicolet Avatar, Thermo Fisher Scientific, Waltham, MA, USA).

### Differentiation of hEnSCs into motor neuron-like cells within 3D collagen hydrogels containing EpoB-loaded microspheres

Differentiation of hEnSCs was conducted in a three-step approach^26^ using 24-well cell culture plates with different experimental groups, including collagen hydrogel without Mic EpoB (2-Col/PA-CH), collagen hydrogel with Mic EpoB (2-Col/PA-CH/Mic EpoB), tissue culture plate (TCP; hEnSCs differentiation without Mic EpoB and collagen hydrogel), and control (undifferentiated hEnSCs). 2 × 10^5^ hEnSCs in complete medium (DMEM/F12 and 10% FBS) at passage three were seeded on different substrates and incubated at 37 °C and 5% CO_2_. After 24 h, in step one, the complete medium was replaced with a pre-induction (PrI) medium, and cells were incubated for 24 h. In step two, the PrI medium was exchanged with the induction medium, and cells were incubated for 7 days (the medium was exchanged after 3 days). In step three, the induction medium was replaced with a maturation medium, and incubation was continued for an additional 7 days (the medium was exchanged after 3 days). All procedures and reagents are summarized in Table 3.

**Table 3:** The procedure and reagents used for differentiation of hEnSCs into motor neuron-like cells on 3D collagen hydrogels with or without EpoB-loaded PCL microspheres or cell culture plates.

Step	Differentiation medium	Ingredients	Catalog number	Duration
1	Pre-induction (PrI) medium	DMEM/F12 (1:1)	Invitrogen, 32500-035	1 days
		20% FBS	Gibco, 10270-106	
		2% B-27 supplement	Gibco, 17504-044	
		100 μM 2-Mercaptoethanol	Sigma, M3148	
		250 μM isobutylmethylxanthine (IBME)	Sigma, I5879	
		10 ng/mL fibroblast growth factor 2 (FGF2)	Sigma, SRP4037	
2	Induction medium (exchanged after 3 days)	DMEM/F12 (1:1)	Invitrogen, 32500-035	7 days
		0.2% B-27 supplement	Gibco 17504-044	
		100 ng/mL sonic hedgehog (SHH)	Sigma, SRP3156	
		0.01 μM retinoic acid (RA)	Sigma, R2625	
3	Maturation (survival) medium (exchanged after 3 days)	DMEM/F12 (1:1)	Invitrogen, 32500-035	7 days
		0.2% B-27 supplement	Gibco 17504-044	
		100 ng/mL glial cell-derived neurotrophic factor (GDNF)	Sigma, G1777	
		200 ng/mL brain-derived neurotrophic factor (BDNF)	Sigma, SRP3014	

### Immunofluorescence (IF) staining

All samples were fixed using 4% paraformaldehyde in PBS for 30 min at room temperature and then permeabilized using 0.2% Triton X-100/PBS. The cells were blocked using 5% BSA/PBS for 45 min at room temperature and incubated overnight with primary antibodies at 4 °C (Table 4). Then, each sample was washed three times with PBS/Tween 20 (0.1%, Sigma). Secondary antibodies were added, and samples were kept in the dark for one hour at room temperature. Cells were again washed with PBS/Tween 20 (0.1%) three times. Cell nuclei were counterstained with 4′,6-diamidino-2-phenylindole (DAPI; Sigma-Aldrich, D8417). For the negative control group, only secondary antibodies were used.

**Table 4:** Primary and secondary antibodies were used for immunofluorescence staining.

Antibody/stain	Catalog number	Dilution
**Primary antibody**		
Anti-beta-III-tubulin antibody (Tuj-1) (rabbit monoclonal)	Abcam, ab68193	1:200
Anti-Islet-1 (mouse monoclonal)	Abcam, ab86472	1:200
Anti-choline acetyltransferase (CHAT; mouse monoclonal)	Santa Cruz, sc-55557	1:200
Anti-nestin (NES; mouse monoclonal)	PadzaCo, MN105	1:200
Anti-Homeobox 9 (HB9; mouse monoclonal)	Santa Cruz, sc-515769	1:50
**Secondary antibody**		
Goat anti-rabbit IgG (H + L) cross-adsorbed secondary antibody, Alexa Fluor 488	Gibco, A-11008	1:500
Goat anti-mouse IgG (H + L) highly cross-adsorbed secondary antibody Alexa Fluor 594	Gibco, A-11032	1:700
**Nuclear stain**		
4′,6-Diamidino-2-phenylindole (DAPI)	Sigma-Aldrich, D8417	1:1000

### RNA extraction and cDNA synthesis

Total RNA extraction was conducted using RNX Plus™ low copy RNA isolation (CinnaGen, Iran, EX6101), chloroform, isopropyl alcohol, and 75% ethanol. The purity and quantity of each extracted RNA were assessed using a Nanodrop spectrophotometer (Thermo Scientific, USA). cDNA synthesizing was conducted using PrimeScript™ RT Reagent Kit (Takara, RR037A) following the manufacturer’s instruction (Thermocycler schedule: 15 min at 37 °C, 5 s at 85 °C).

### Real-time RT-PCR for gene expression analysis

**Figure 1:**
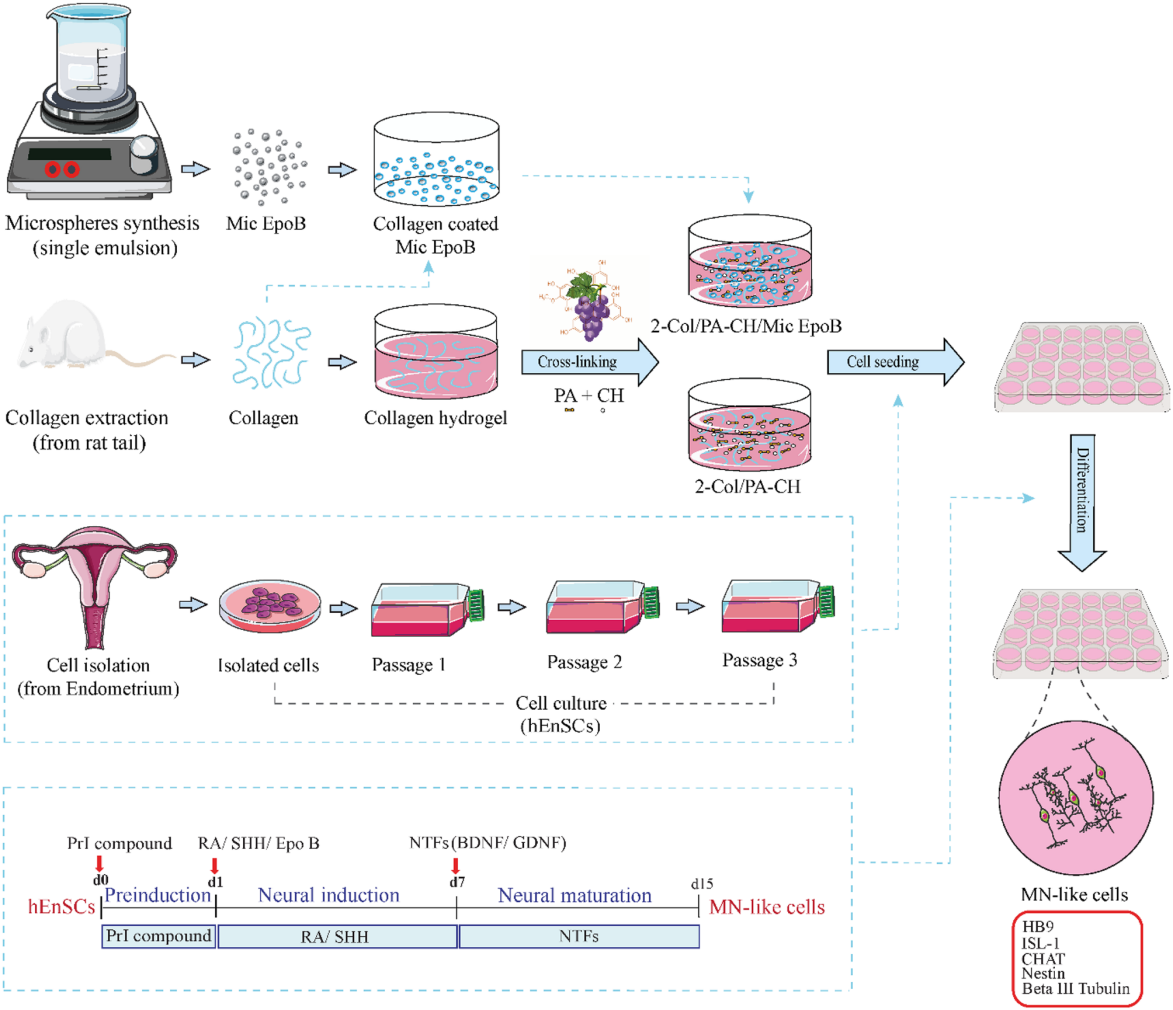
A schematic presentation of different steps of the study, including; EpoB microspheres synthesis using single emulsion, collagen extraction from rat tail, synthesis of collagen hydrogel and its crosslinking using proanthocyanidin from grape seed and calcium hydroxide (PA-CH), cell isolation, and culturing from human endometrium, and a three-step MN-differentiation (during 15 days).

All primers were obtained from Qiagen (Germany) (Table 5). RealQ RNA quantitative measurement was evaluated using Plus 2 × Master Mix Green (AMPLIQON, 5000850-1250) on a real-time thermocycler (Rotor-Ge4ne Q, Corbett Life Science, USA) (Table 6). PCR product specificity was evaluated by confirming a single peak in the melting curve analysis. All experimental samples were performed three times in duplicate. *RPL13A* was selected as a housekeeping gene, and each gene fold-change was calculated relative to *RPL13A* based on relative quantitation evaluation via the ΔΔCT method, using the 2^−ΔΔCT^ relative expression equation. A summary of the different steps of the study is shown in Fig. 1.

**Table 5:** Primers used for real-time RT-PCR.

Gene	Assay name	Catalog number (Qiagen)	Genebank accession number	Amplicon length (bp)
NES [Human]	Hs_NES_2_SG QuantiTect Primer Assay	QT01015301	NM_006617	75
ISL1 [Human]	Hs_ISL1_1_SG QuantiTect Primer Assay	QT00000294	NM_002202	83
NEFH [Human]	Hs_NEFH_1_SG QuantiTect Primer Assay	QT00209181	NM_021076	97
MNX1 [Human]	Hs_MNX1_vb.1_SG QuantiTect Primer Assay	QT02407384	NM_005515	130
RPL13A [Human]	Hs_RPL13A_1_SG QuantiTect Primer Assay	QT00089915	NM_012423	161

**Table 6:** Reaction conditions of real-time PCR.

Reaction detail		Real-time PCR schedule		
Component	Volume	Step	Temperature	Time
SYBR Green I Master Mix	5 µL	Initial denaturation	95 °C	15 min
Diluted cDNA (1:10)	1 μL	Denaturation at	94 °C	15 s
Related primer	1 μL	Annealing temperature	60 °C	30 s
dH2O	3 µL	Extension	72 °C	30 s
	Total 10 µL

### Analysis and quantification of neurite extension

Analysis of neurite extension was investigated using an inverted phase-contrast microscope (Olympus, Japan) attached to a digital camera (DP50). For each experimental group, five random representative areas were selected for neurite length measurement using ImageJ software.

### Statistical data analysis

Data were analyzed using SPSS 21 software. One-way ANOVA and Dunnett’s two-tailed t-test (DUNNETT) were employed to evaluate the statistical significance of differences between the control and all experimental groups.

### Ethics declarations

Ethical approval for collecting biopsy disposal of patients human endometrial was obtained from the Ethics Committee of Tehran University of Medical Sciences (code: IR.TUMS.REC.1394.1137). An informed consent was obtained from patients/legal guardians according to Tehran University of Medical Sciences guidelines. All methods were performed in accordance with the relevant guidelines and regulations.

## Results

### Characterization of hEnSCs derived from endometrium

**Figure 2:**
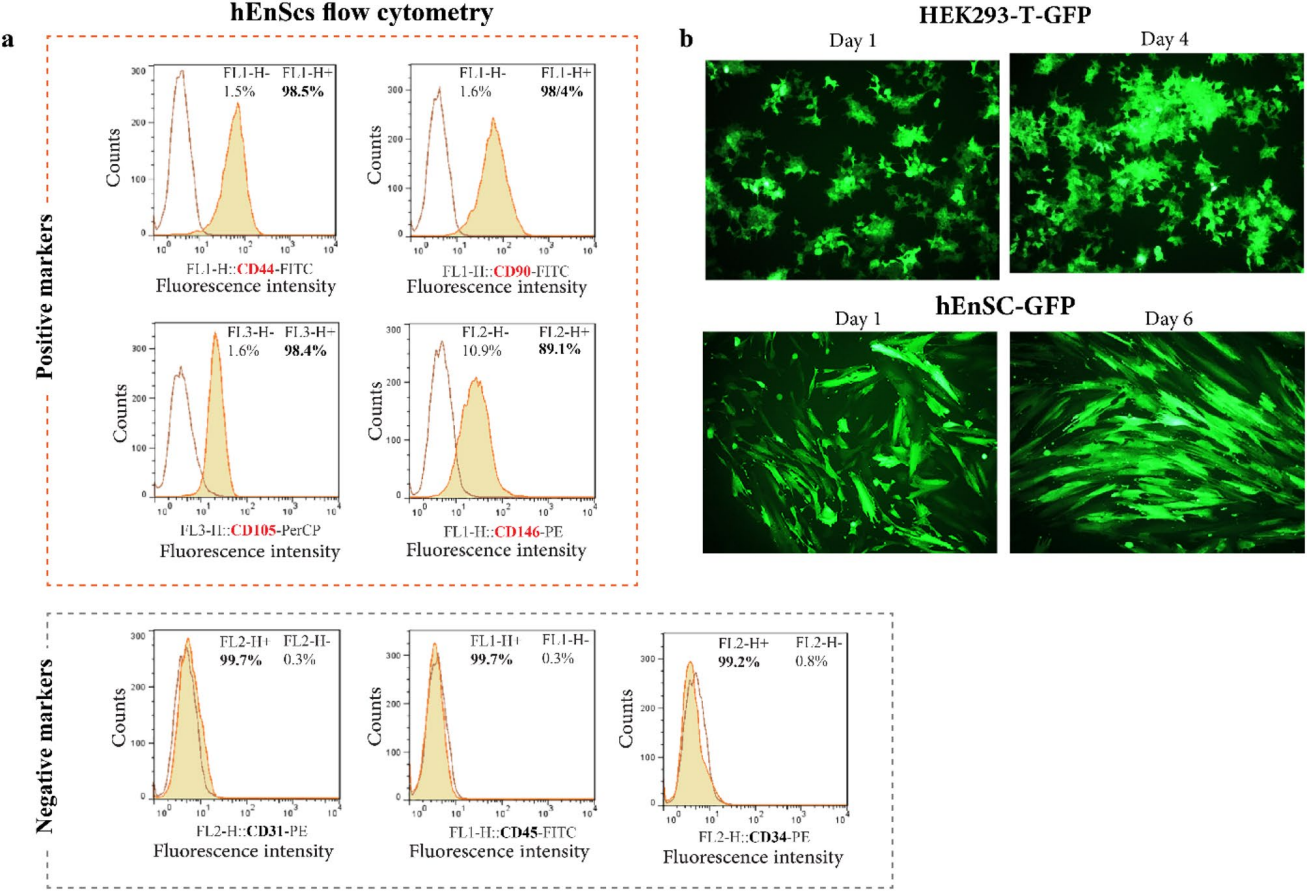
Characterization of cell markers for hEnSCs and verification of GFP-labeling. (**a**) The results of flow cytometric assessment of hEnSCs at passage three, isolated from the endometrial biopsy. Dark red lines display isotype control (negative control). hEnSCs were positive for mesenchymal stem cell markers CD44 (98.5%), CD90 (98.4%), and CD105 (98.4%), endometrial stem cell marker CD146 (89.1%), shown as a right shift of the fluorescent intensity peak. hEnSCs were also negative for hematopoietic CD45, CD34, and endothelial CD31 markers (no shift of the fluorescent peak was observed). (**b**) HEK293-T and hEnSCs transduction with GFP-expressing lentiviral vectors. To confirm GFP expression, the labeled HEK293-T and hEnSCs were monitored with a fluorescent microscopy.

The hEnSCs were positive for mesenchymal stem cell markers CD44 (98.5%), CD90 (98.4%) and CD105 (98.4%), endometrial stem cell marker CD146 (89.1%). hEnSCs were also negative for hematopoietic CD45, CD34, and endothelial CD31 markers (Fig. 2a).

### Cell GFP transduction

Two types of cells, HEK293-T, and hEnSCs were labeled. As shown in Fig. 2b, cells were effectively transduced by GFP-encoding lentiviral particles. The GFP-positive cells were observed by fluorescent phase-contrast microscopy and used to assess cell adhesion and proliferation within hydrogels.

### EpoB-loaded PCL microsphere characterization

#### Scanning electron microscope

**Figure 3:**
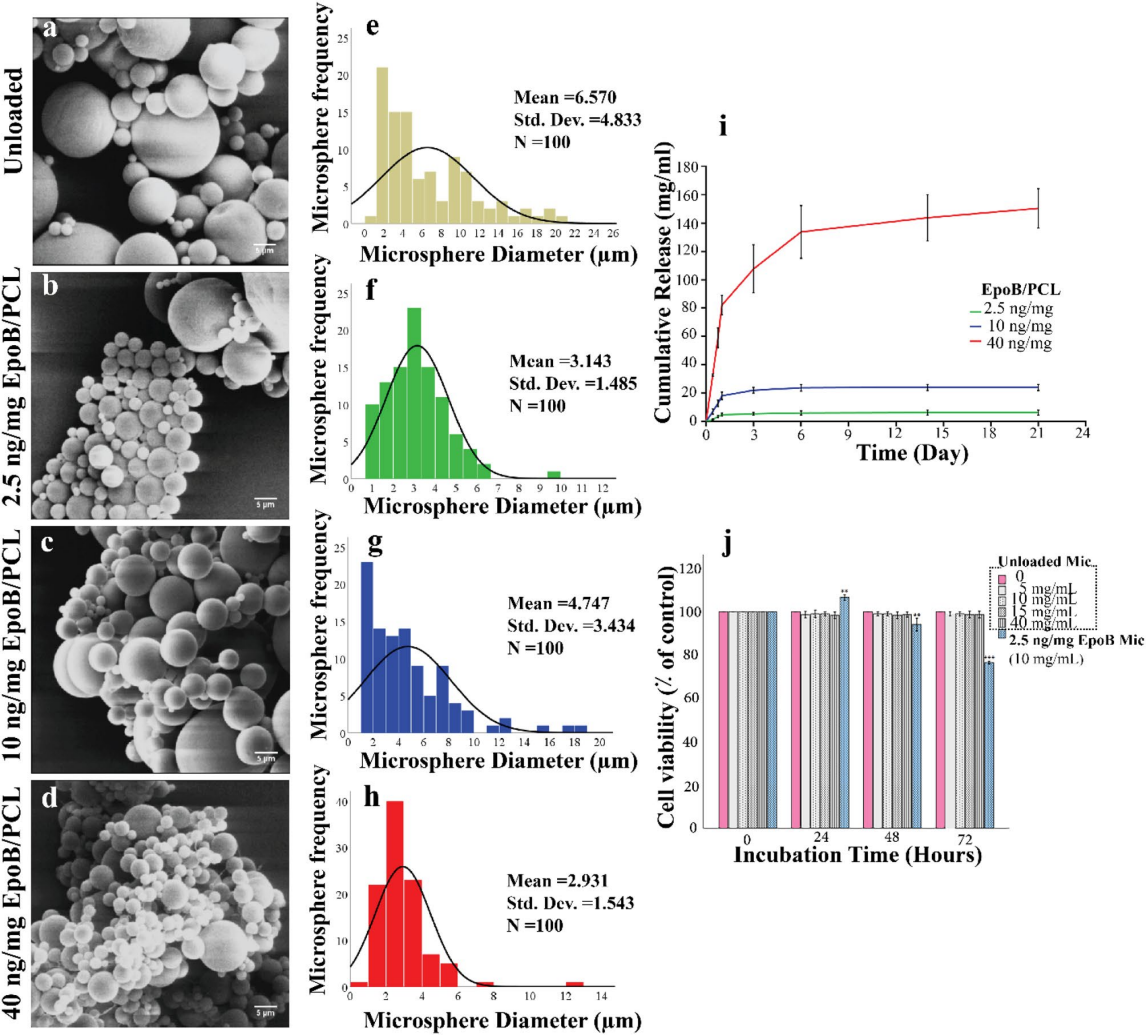
Characterization of microspheres. Scanning electron microscopy (SEM) and characterization of unloaded, 2.5, 10, and 40 ng/mg EpoB/PCL microspheres. (**a**–**d**) SEM shows smooth morphology and some microsphere clustering. (**e**–**h**) Particle size distribution histogram indicating expected particle size diameters (Sample size n = 100). (**i**) The cumulative release profile of 2.5, 10, and 40 ng/mg EpoB/PCL microspheres over 21 days (7-time points). Sample size n = 3. (**j**) The effect of 2.5 ng/mg EpoB Mic and different concentrations of PCL microspheres (unloaded Mic) on cell viability of hEnScs. Data are displayed as the percentage of cell viability in three independent tests, with each test having three individual samples. Cell viability graphs were compiled using SPSS 21 (clustered bar, summaries for groups of cases). (Error bars: ± 1 SD). P values were assessed with SPSS 21, using one-way ANOVA and DUNNETT test. The effect of all samples was compared to 0 (control). *P < 0.05, **P < 0.01, ***P < 0.001.

The morphology of the fabricated microspheres was assessed using SEM. SEM images indicated a round shape with some clustering and smooth surface. The diameters of 2.5, 10, and 40 ng/mg EpoB/PCL microspheres were 3.143 ± 1.485 µm, 4.747 ± 3.434 µm, and 2.931 ± 1.543 µm, respectively. Compared to the EpoB/PCL microspheres, the unloaded microspheres were larger, with a diameter of 6.570 ± 4.833 (P < 0.001) (Fig. 3a–h).

#### Encapsulation efficiency

The encapsulation efficiencies (EE) for different loading concentrations of EpoB are shown in Table 7. A greater EE was obtained in PCL microspheres with higher EpoB loading levels, indicating that the initial concentration of EpoB can potentially affect EE of EpoB in PCL microspheres using the single emulsion fabrication method.

**Table 7:** Encapsulation efficiencies for different drug loading.

EpoB concentration added	Encapsulation efficiency ± SD (%)
2.5 ng/mg EpoB/PCL	83 ± 1
10 ng/mg EpoB/PCL	88.9 ± 0.5
40 ng/mg EpoB/PCL	93.9 ± 0.3

Sample size n = 3, P < 0.001.

#### EpoB release

As shown in Fig. 3i, there was a burst release in the initial first 24 h for all groups, followed by a slower gradual release for 2.5 and 10 ng/mg EpoB/PCL microspheres, which reached a plateau. However, compared to 2.5 and 10 ng/mg EpoB/PCL microspheres, the 40 ng/mg EpoB/PCL microspheres maintained a higher release rate over 21 days, indicating that the release rate is influenced by the amount of EpoB encapsulated in the microspheres. According to the EpoB release results, 2.5 ng/mg was selected due to its appropriate sustained release dosage for neuronal differentiation.

### Proliferation and viability assessment of microspheres

To find the permissible limit, the effect of four different concentrations of unloaded PCL microspheres (unloaded Mic: 5, 10, 15, and 40 mg/mL) were assessed for viability and proliferation of hEnSCs during 24, 48, and 72 h using the MTT assay. The effect of the candidate microsphere 2.5 ng/mg (Mic EpoB) on hEnSCs was also evaluated by indirect MTT assay for 24, 48, and 72 h (Fig. 3j). The results indicated no cytotoxicity in all four PCL microsphere concentrations. For Mic EpoB, cell proliferation increased after 24 h. The Mic EpoB cell viability at 48 and 72 h after treatment was 94.1% (P < 0.01) and 76.5% (P < 0.001), respectively.

### Cell morphology, attachment, and proliferation

#### Fluorescent microscopy

**Figure 4:**
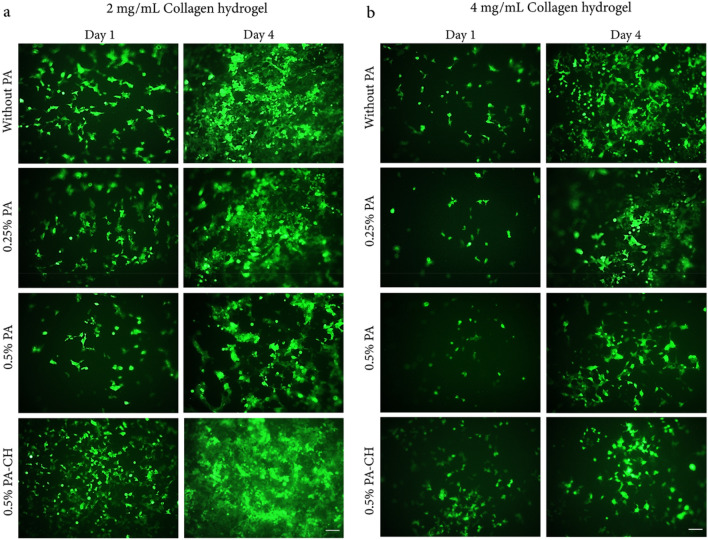
HEK293-T-GFP morphology, adhesion, and proliferation within collagen hydrogels. The HEK293-T-GFP adhered to (**a**) 2 mg/mL and (**b**) 4 mg/mL collagen hydrogel concentrations and all cross-linked collagen hydrogels (using 0.25% PA, 0.5% PA and 0.5% PA-CH). Over time, more GFP-positive cells were observed, indicating that the proliferated cells were GFP positive, as well. Cell proliferation was more prominent in hydrogels fabricated using 2 mg/mL collagen.

**Figure 5:**
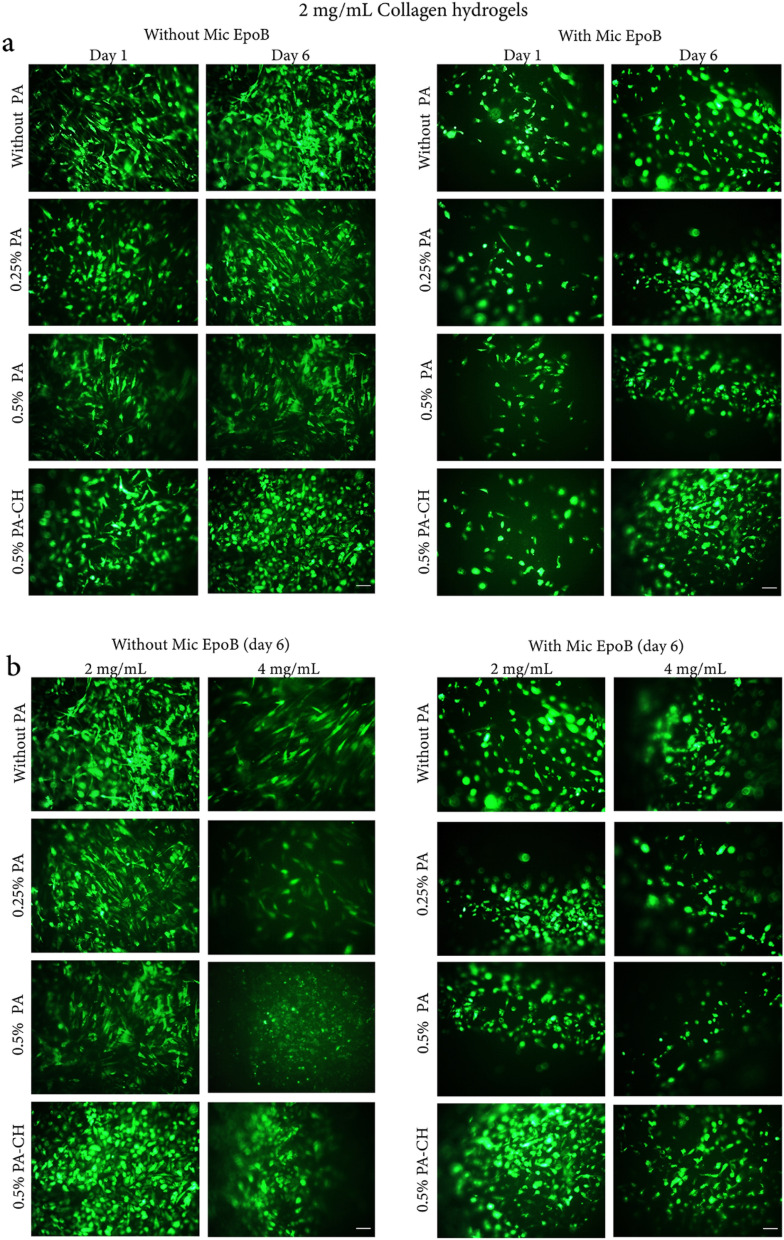
Adhesion and proliferation of hESCs-GFP within collagen hydrogels before and after cross-linking using different concentrations of PA (0.25% PA, 0.5% PA, and 0.5% PA-CH). (**a**) Collagen hydrogels were fabricated using 2 mg/mL collagen with or without Mic EpoB at day 1 and day 6, (**b**) and a comparison between hydrogels was performed using different collagen concentrations (i.e., 2 mg/mL and 4 mg/mL) with or without Mic EpoB at day 6 of culture.

Collagen hydrogels improved adhesion and proliferation of HEK293-T (as a typical well-transected GFP cell) and hEnSCs-GFP. As shown in Figs. 4 and 5, the effect of hydrogel fabricated using 2 mg/mL collagen was more prominent on cell proliferation compared with hydrogel fabrication using 4 mg/mL collagen. Furthermore, among different groups, the 0.5% PA-CH hydrogel had the greatest cell proliferation compared to other PA-crosslinked collagen hydrogels in both 2 mg/mL and 4 mg/mL collagen concentrations (i.e., 2-Col/PA-CH and 4-Col/PA-CH). Since the 2-Col/PA-CH hydrogel had a more prominent effect on cell proliferation compared to 4-Col/PA-CH, this formulation was selected as the optimized hydrogel for further experiments.

#### Scanning electron microscope (SEM)

**Figure 6:**
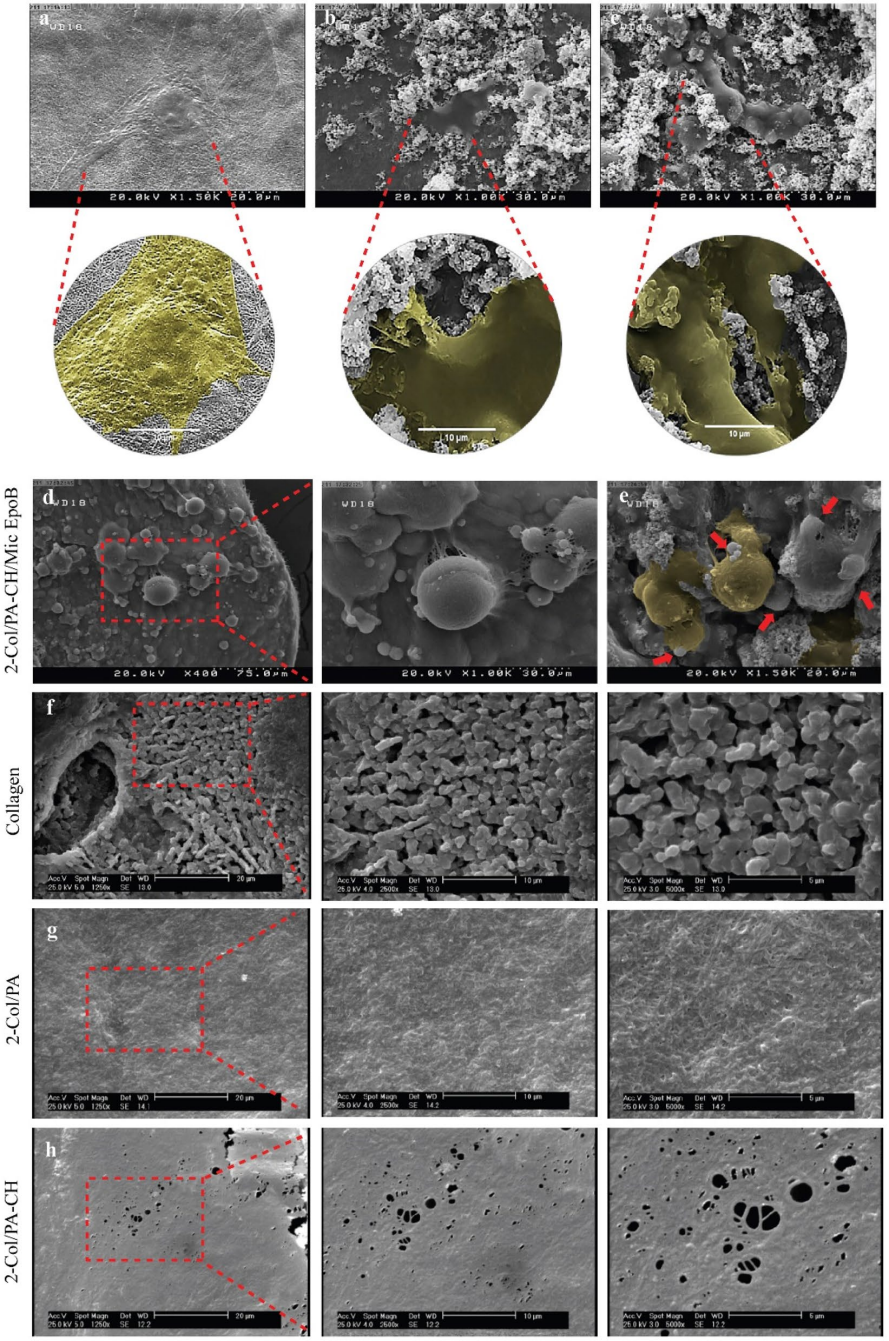
Cell attachment and surface morphology of different collagen hydrogel groups. Scanning electron micrographs of hEnSCs attachment on (**a**) collagen, (**b**) 2-Col/PA-CH, and (**c**) 2-Col/PA-CH/Mic EpoB hydrogels 48 h after cell seeding. Micrographs show hydrogel with cells at × 1000 and × 3000 magnifications (pseudo-colored). Scanning electron micrographs of different collagen hydrogel groups (**d**–**h**) indicates their surface morphology and interconnected porosity: 2-Col/PA-CH/Mic EpoB (**d**,**e**), collagen (**f**), 2-Col/PA (**g**), and 2-Col/PA-CH (**h**). EpoB microspheres were detected in the 2-Col/PA-CH/Mic EpoB hydrogel (**d**,**e**). Moreover, the cell attachment (highlighted in yellow) and EpoB microspheres (pointed with red arrows) are displayed in 2-Col/PA-CH/Mic EpoB hydrogel in Figure **e**.

SEM images of hEnSCs cultured on collagen, 2-Col/PA-CH, and 2-Col/PA-CH/Mic EpoB hydrogels indicated cell-scaffold interactions and cell attachment on hydrogel scaffolds 48 h after cell seeding (Fig. 6a–c,e). The interconnected porosity and surface morphology of the hydrogel scaffolds was revealed by SEM. In the 2-Col/PA-CH/Mic EpoB hydrogel, the EpoB microspheres were visible and detected (Fig. 6d,e). The 2-Col/PA and 2-Col/PA-CH hydrogels showed more fibrous and regular structure compared to collagen (Fig. 6f–h), and the 2-Col/PA-CH hydrogel indicated a smooth surface morphology.

### Hydrogel biodegradation

**Figure 7:**
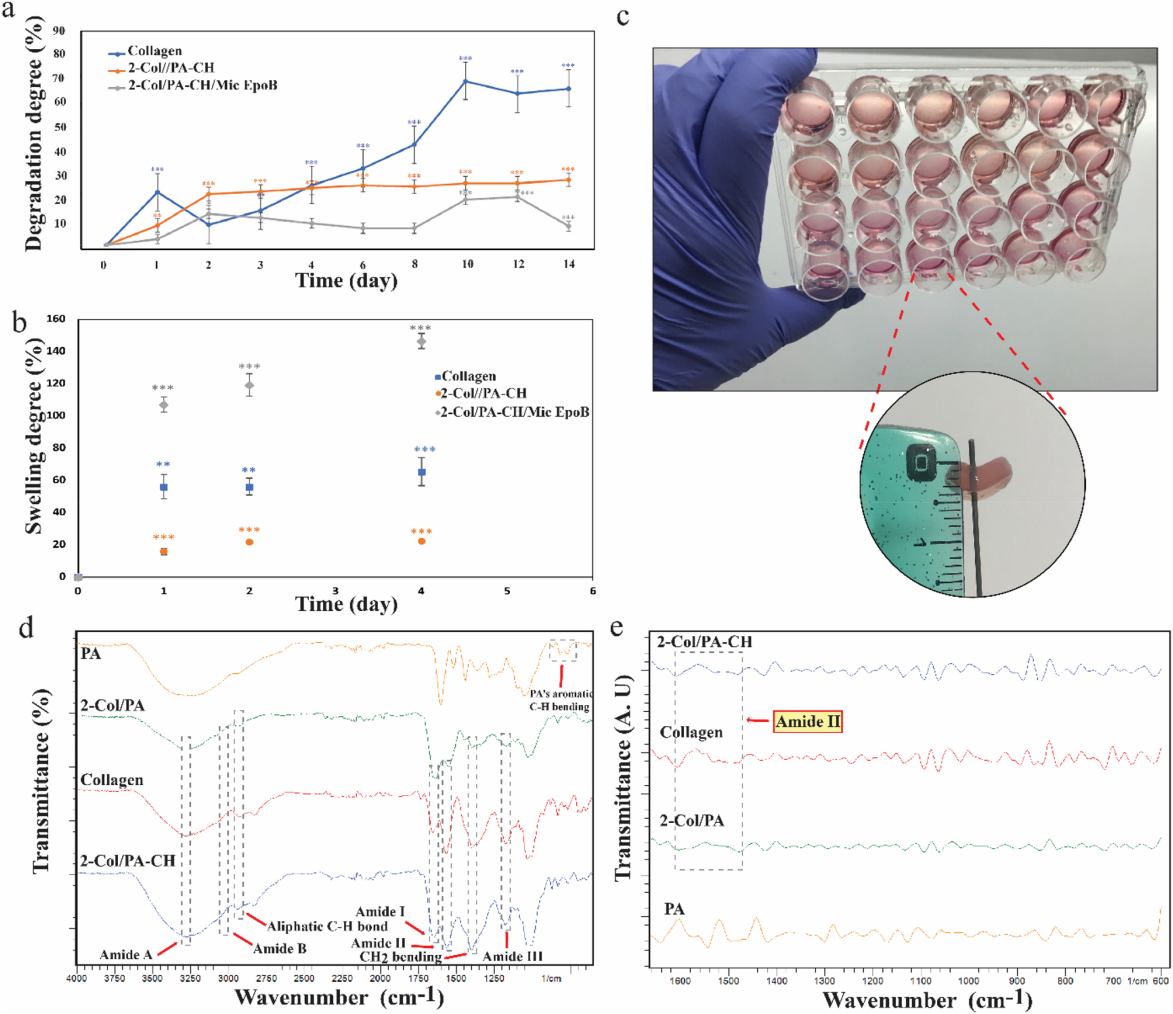
Degradation and swelling degree, and ATR-FTIR spectra of different collagen hydrogel groups. (**a**) Degradation and (**b**) swelling degree of collagen, 2-Col/PA-CH (Cross-linked 2 mg/collagen hydrogel with 0.5% proanthocyanidin and calcium hydroxide), and 2-Col/PA-CH/Mic EpoB (Cross-linked 2 mg/mL collagen hydrogel with 0.5% proanthocyanidin and calcium hydroxide and 2.5 ng/mL Epo B microspheres) hydrogels in DEME-F12 at 37 °C (The time duration: 14 days for degradation test and 4 days for swelling test). *P < 0.05, **P < 0.01, ***P < 0.001. (**c**) The consistency in cross-linked collagen hydrogels. (**d**) The ATR-FTIR spectra of PA, collagen, 2-Col/PA, 2-Col/PA-CH and, (**e**) their second-derivative spectra in the amide B region. The main peaks are shown by red arrows and gray dash lines. The second-derivative spectra in the amide B region indicate secondary structure conformational changes in the two cross-linked hydrogels scaffold (2-Col/PA and 2-Col/PA-CH) compared to the collagen (uncross-linked) hydrogel scaffold.

The degradation of different hydrogel formulations, including collagen, 2-Col/PA-CH, and 2-Col/PA-CH/Mic EpoB, during 14 days of incubation in DMEM-F12 medium at 37 °C was evaluated (Fig. 7a). The degradation degree of collagen hydrogel was higher than the other two groups (i.e., 2-Col/PA-CH, and 2-Col/PA-CH/Mic EpoB). Adding of collagen-coated Mic EpoB and PA-CH as a cross-linker increased the stability of the collagen hydrogel. Furthermore, 2-Col/PA-CH/Mic EpoB showed slower degradation and higher stability compared with 2-Col/PA-CH.

### Hydrogel swelling

The swelling degree of collagen, 2-Col/PA-CH, and 2-Col/PA-CH/Mic Epo B hydrogels in DMEM-F12 was calculated until 96 h (Fig. 7b). The swelling degrees were as follow:

$$2{\text{-Col/PA-CH/Mic}}\;{\text{Epo }} > {\text{ collagen }} > {\text{ 2-Col/PA-CH}}.$$

The results showed that adding PA-CH as cross-linker causes a decrease in the swelling degree of collagen. However, the addition of coated collagen microspheres to collagen gel increased the swelling degree of the hydrogel. The consistency in cross-linked collagen hydrogels is presented in Fig. 7c.

### ATR-FTIR spectroscopy

The ATR-FTIR spectra of PA, collagen, 2-Col/PA, 2-Col/PA-CH are presented in Fig. 7d. The main peaks including amide A (3280–3290 cm^−1^), amide B (3075–3085 cm^−1^), amide I (1635–1656 cm^−1^), amide II (1549–1566 cm^−1^), amide III (1174–1177 cm^−1^), aliphatic C–H bond (2925–2942 cm^−1^), CH_2_ bending (1397–1401 cm^−1^) were detected and pointed out by arrows and dash lines. Additionally, to enhances the separation of overlapping peaks, second-derivative spectrum in the amide B region was achieved via irAnalyze software, showing secondary structure conformational changes in the two cross-linked hydrogels scaffold (2-Col/PA and 2-Col/PA-CH) compared to the collagen (uncross-linked) hydrogel scaffold (Fig. 7e).

### The effect of 3D collagen hydrogel and EpoB release on differentiation of hEnSCs into motor neuron-like cells

#### Immunofluorescence staining

**Figure 8:**
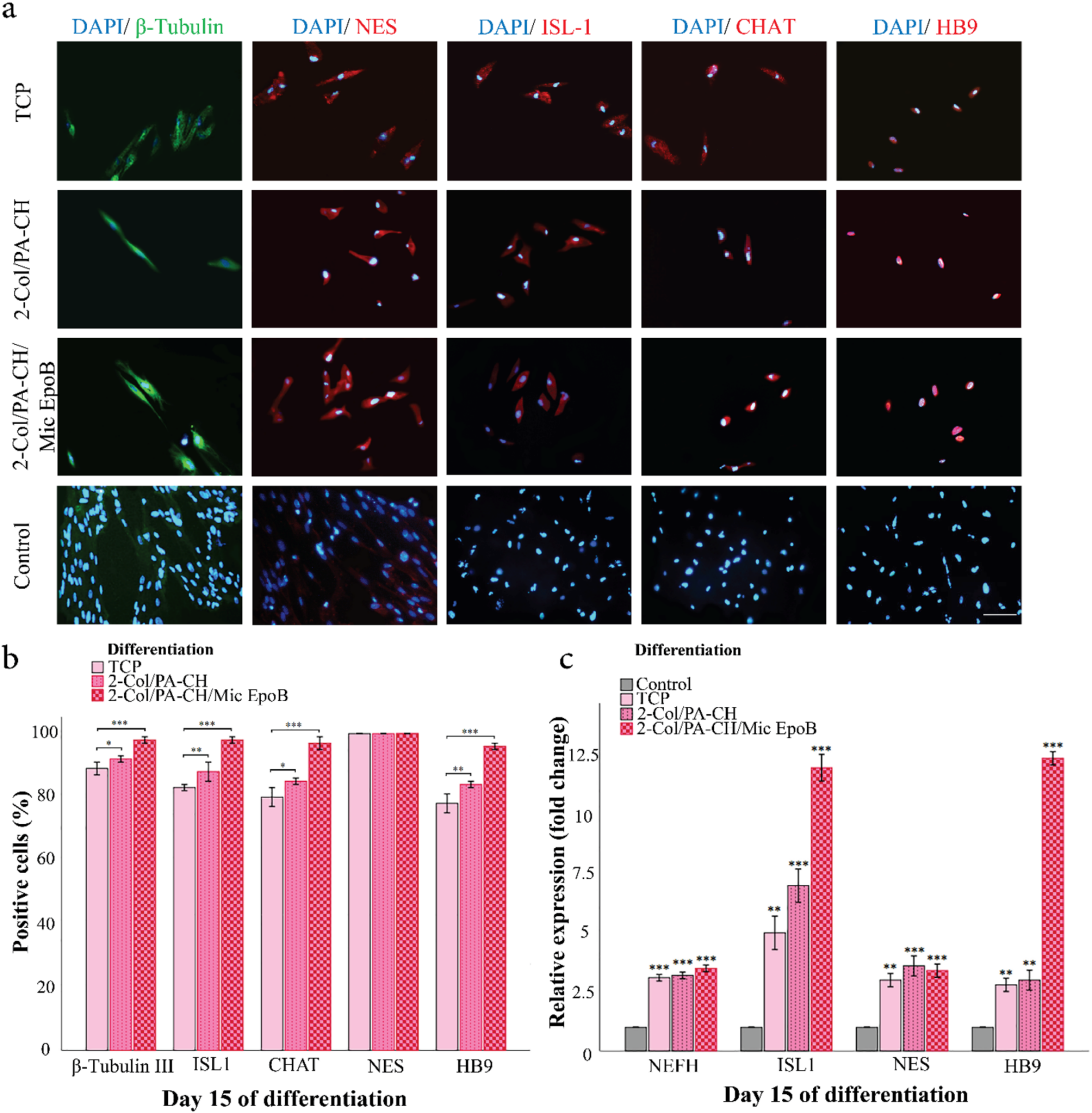
The expression of MN-like cells differentiated from hEnSCs in different groups. (**a**) Immunofluorescence images of MN-like differentiated hEnSCs in different groups of TCP, collagen hydrogel without Mic EpoB (2-Col/PA-CH/M), collagen hydrogel with Mic EpoB (2-Col/PA-CH/Mic EpoB), and control (undifferentiated hEnSCs) for beta-III-tubulin, NES, ISL-1, CHAT, and HB9 after 15 days (× 20, Scale bar 100 μm). (**b**) Expression (ratio of positive cells, %) of MN markers calculated from three wells (five fields per well). (**c**) Relative gene expression in three differentiated groups, including TCP, Col without Mic EpoB, Col with Mic EpoB, and control (undifferentiated hEnSCs) after 15 days for NEFH, ISL-1, NES, and HB9. Graphs were generated with SPSS 21 (simple bar, summaries for groups of cases). Data were presented as three independent experiments. (Error bars: ± 1 SD). The p-values were assessed using SPSS 21, one-way ANOVA, and the DUNNETT test. In part B: Col without Mic EpoB and Col with Mic EpoB groups were compared with TCP: *P < 0.05, **P < 0.01, ***P < 0.001. In part C: The effects of three differentiation groups (TCP, col-without Mic EpoB, and col-with Mic EpoB) compared with control group (undifferentiated hEnSCs): *P < 0.05, **P < 0.01, ***P < 0.001.

After 15 days of MN-induction, the morphology of hEnSCs changed from a fibroblast-like shape to a bipolar and round cell body shape with neurites. The neuron-like morphology was observed using an inverted phase-contrast microscope (Olympus, Japan). IF staining results showed up-regulation of the MN markers ISL-1, CHAT, and HB9 in all three experimental groups, but no expression in the control group (Fig. 8a).

Differentiation rate was investigated by counting each positive cell marker (beta-tubulin III, ISL-1, CHAT, NES, HB9) as a percentage of the total number of DAPI-stained cells. The level of positive MN markers expression in cells seeded on 2-Col/PA-CH/Mic EpoB group was significantly higher (P < 0.001) than the other two groups (i.e., TCP, and 2-Col/PA-CH), except for NES. The positive marker expression of different evaluated markers in TCP, 2-Col/PA-CH, and 2-Col/PA-CH/Mic EpoB is shown below, respectively. Quantitative results are also shown in Fig. 8b.

- beta-tubulin III (89%, 92%, 98%,)
- ISL-1 (83%, 88%, 98%)
- CHAT (80%, 85%, 97%)
- NES (100%, 100%, 100%)
- HB9 (78%, 84%, 96%)

#### Molecular analysis using real-time-PCR

After 15 days of cell induction, real-time RT-PCR was performed to compare the fold-change of RNA expressions of *HB9*, *ISL-1*, *NES*, *and NEFH* in three experimental groups, including TCP, 2-Col/PA-CH, 2-Col/PA-CH/Mic EpoB and control (undifferentiated hEnSCs) (Fig. 8c). The real-time-RT-PCR results indicated that the level of *HB9*, *ISL-1*, *NES*, *and NEFH* was significantly up-regulated in all three induced hEnSCs groups (P < 0.001, P < 0.01). Between the three experimental groups, the 2-Col/PA-CH/Mic EpoB hydrogel had the highest expression level of *HB9* and *ISL-1* as MN markers (12.4- and 12-fold change, respectively). The expression of *HB9* in the 2-Col/PA-CH/Mic EpoB hydrogel was 4.43- and 4.13-times higher than the TCP and 2-Col/PA-CH hydrogel, respectively (2.8- and 3-fold change, respectively). The expression of *ISL-1* in the 2-Col/PA-CH/Mic EpoB hydrogel was 2.4- and 1.71-times higher than the TCP and 2-Col/PA-CH, respectively (5- and 7-fold change, respectively). The *NES* and *NEFH* expressions in the 2-Col/PA-CH/Mic EpoB hydrogel were almost the same as TCP and 2-Col/PA-CH groups.

#### Neurite extension

**Figure 9:**
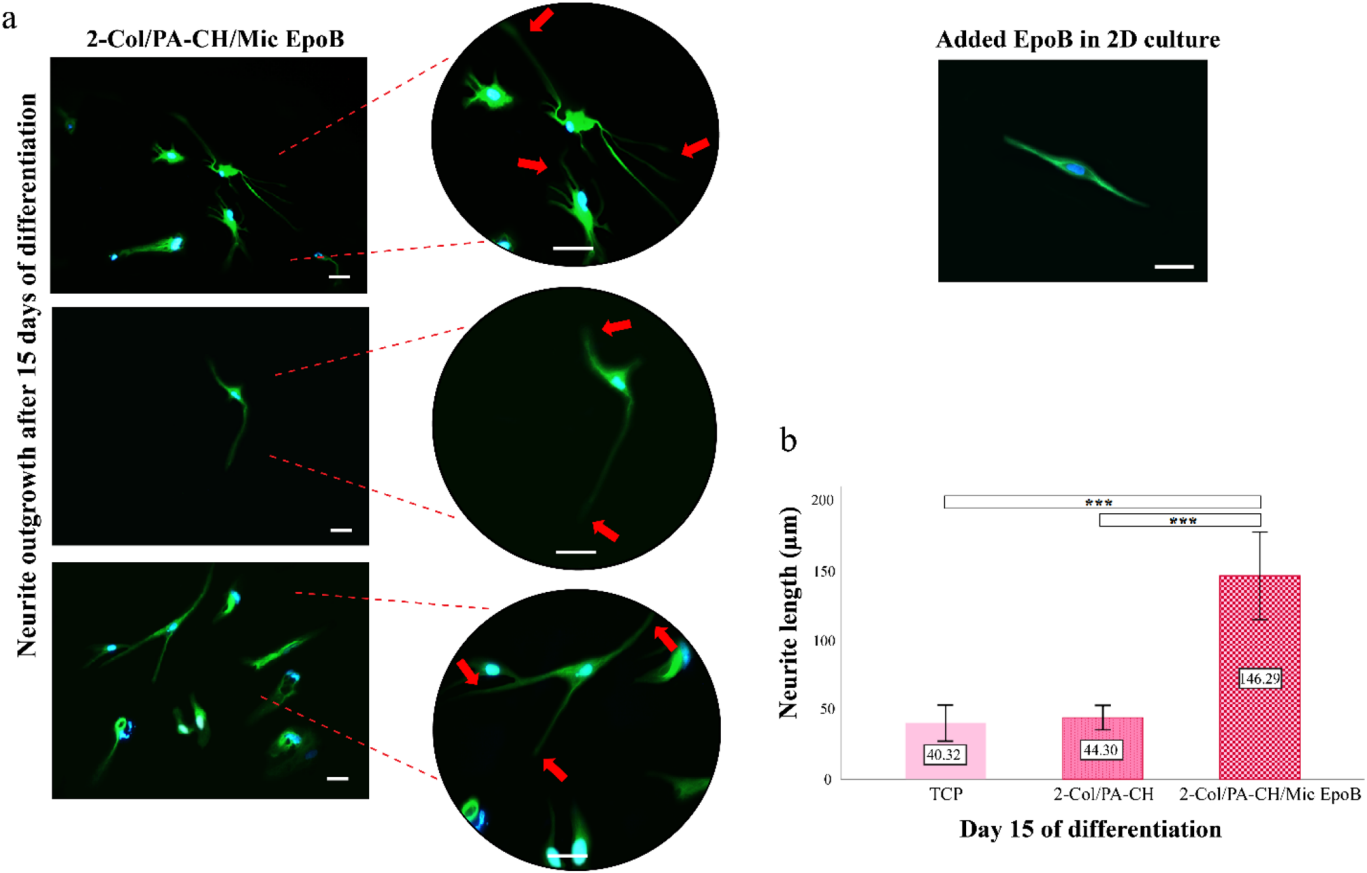
Neuronal morphology and neurite outgrowth. (**a**) Immunofluorescence images indicate neuronal morphology and neurite elongation of MN-like differentiated hEnSCs after 15 days of induction in collagen hydrogel with Mic EpoB (2-Col/PA-CH/Mic EpoB) group. (**b**) Neurite elongation is shown with red arrows. To display morphology, cells were stained using primary anti-beta III tubulin antibody and secondary Alexa Fluor 488 antibody (× 20 and × 40; Scale bar 100 μm). Neurite length of MN-like cells in 2-Col/PA-CH/Mic EpoB hydrogel compared to TCP and 2-Col/PA-CH differentiation groups at day 15 after differentiation (***P < 0.001).

The neurite length of the MN-like cells was significantly longer in 2-Col/PA-CH/Mic EpoB hydrogels compared to TCP and 2-Col/PA-CH groups (P < 0.001) after 15 days of MN-induction (Fig. 9).

## Discussion

In recent years, increasing evidence suggests that small molecules are effective chemical agents for cell survival and differentiation, and may be candidates for various interventional therapies^60^. Small molecules often regulate the activity of several transcription factors, which can thereby determine cell fate. Thus, exposing stem cells to small molecules can advance cell differentiation by mimicking tissue microenvironment during organogenesis^61,62^. Ruschel et al. reported low doses of EpoB, known as a small molecule, diminishes fibrotic scar tissue and induces axonal regeneration in vitro in injured cortical neurons of postnatal rats^37^. This study aimed to evaluate the effect of sustained release of EpoB using PCL microspheres embedded in 3D collagen hydrogel for differentiation of hEnSCs into MN-like cells.

Many studies have proposed tissue engineering as a practical approach to repairing various damaged tissues, and nerve tissue engineering is no exception to this rule, especially given the increasing rate of SCI^23,63^. Many combinational approaches have proven advantageous compared with using each method separately. However, these approaches require further elucidation to ensure a meaningful effect compared to their individual usage^12^.

It is known that ECMs have active roles in cell survival, neuronal differentiation, and neurite development^12^. Embedding hydrogels alone without any treatment may slightly improve injury outcomes after SCI^8^. However, for the hydrogel approach, it has been shown drug incorporation into hydrogels is necessary for effective healing of the injured environment^8,40^.

In this study, we synthesized EpoB/PCL microspheres to have a sustained release during the MN differentiation process. The results indicated that PCL microspheres mediated the sustained release of EpoB as a microtubule-stabilizing small molecule that enhances differentiation of hEnSCs to MN-like cells and increases axonal elongation. The addition of released EpoB, along with the differentiation protocol, promoted neurite outgrowth and neuronal morphology. It also significantly increased the efficiency of motor neuron generation (˃ 96%), and compared to most recent studies, it gained a higher percentage of motor neurons^64,65^.

PCL was chosen for microsphere synthesis because of its good biocompatibility, low biodegradation, and cost-effectiveness^39^. All four groups of EpoB/PCL microspheres (0, 2.5, 10, and 40 ng/mg) were synthesized using a single emulsion technique, and their round shape microspheres with a smooth surface were demonstrated by SEM. Based on the EpoB release test results, the 2.5 ng/mg EpoB/PCL microsphere group was selected for MN differentiation given a more appropriate dose and drug release and normal size distribution. The indirect MTT test also confirmed the suitability of 2.5 ng/mg EpoB/PCL microsphere for our purposes with cell viability ˃ 77% after 72 h.

Recent studies indicate that culturing stem cells in 3D hydrogels improves cell maintenance, expansion, and differentiation by mimicking the native ECM^66,67^. For neuronal replacement therapies, given that neurons are not capable of dividing, stem cell expansion with hydrogel assistance is essential to achieve a suitable cell number before neuronal differentiation^68^.

Cell attachment to the ECM is crucial for cell differentiation and proliferation via cell anchoring and activation of signaling pathways that can lead to tissue regeneration and development^69–71^. Collagen is a key component of the ECM and is extensively used as a scaffold for its effective properties of improving cell attachment, survival, proliferation, differentiation, and migration^72^. One study also reported that collagen might promote higher survival, adhesion, and proliferation of mesenchymal stem cells^73^. These cell processes are regulated by the interaction of collagen with integrins in cell surface receptors^74^. Moreover, collagen has garnered attention as a delivery system for the sustained release of different drugs and chemical agents^75,76^.

GFP cell culture in the two collagen hydrogel concentrations (2 and 4 mg/mL) presented a dose-dependent proliferation of hEnSC. The lower concentration, 2 mg/mL, was selected because of its greater potential for cell proliferation and cell connection, which are important for neural differentiation. In line with our study, a previous report indicated that lower concentrations of collagen are more appropriate for neuronal differentiation and neurite outgrowth^77^.

Moreover, adding either PA-CH or PA to the collagen hydrogel increased its stability during the 15-day differentiation process by decreasing the hydrogel's degradation degree. However, hydrogels cross-linked with PA-CH showed a greater cell proliferation than PA alone. In addition, cross-linking the collage hydrogel also decreased the swelling degree of the hydrogel. Interestingly, the 2-Col/PA-CH/Mic EpoB had a slower degradation degree than the collagen and 2-Col/PA-CH groups, which can be attributed to the interactions between PCL microspheres and the collagen hydrogel. The 2-Col/PA-CH/Mic EpoB had a higher swelling ratio than the collagen and 2-Col/PA-CH hydrogels. All three groups showed interconnected porous, proper cell-scaffold integration and cell attachment.

Immunofluorescence staining indicated that the beta III-tubulin marker was increased in all three differentiation groups (i.e., TCP, 2-Col/PA-CH, and 2-Col/PA-CH/Mic EpoB). In agreement with previous reports, undifferentiated hEnSCs and other sources of human MSCs have a spontaneous expression of this marker^78^, demonstrating that it is possible for MSCs such as hEnSCs to undergo neuronal differentiation. Furthermore, other studies have shown that hEnSCs can induce spinal motor neuron generation during development by up-regulating dopamine^79^. However, compared to the differentiation groups, the undifferentiated hEnSCs in our study had a lower beta III-tubulin expression. hEnSCs also spontaneously express NES. Thus, both NES and beta III-tubulin markers show hEnSCs to be a suitable source for neuronal differentiation and spinal cord restoration.

Real-time RT-PCR determined significant gene up-regulation of *NEFH*, *NES*, *ISL-1*, and *HB9* after 15 days of induction. Higher gene expression of *ISL-1* and *HB9* in the 2-col/PA-CH/MicEpoB group compared to the other two differentiation groups showed that incorporation of EpoB microspheres in the collagen hydrogels may significantly increase the MN differentiation efficacy of hEnSCs.

The results show that the release of EpoB from microspheres, in addition to RA and SHH, increase *HB9* gene expression^80,81^, and can significantly enhance the gene expression of *HB9* and also *ISL-1*, which are two crucial regulators of mature motor neurons^82,83^. Moreover, among the other two groups, the 2-Col/PA-CH group demonstrated higher *ISL-1* gene expression.

The percentage of HB9 and ISL-1 positive cells is known to be an indicator of motor neuron differentiation efficiency^84^. The three differentiation groups, 2-Col/PA-CH/Mic EpoB, 2-Col/PA-CH, and TCP, demonstrated 94%, 84%, and 78% HB9 positive cells, while this was 98%, 88%, and 83% for ISL-1, respectively. This confirms the higher MN-differentiation efficiency of the 2-Col/PA-CH/Mic EpoB group compared to the other two differentiation groups. This is while a previous study indicated 11.7% and 23.7% positive cells for HB9 and ISL-1, respectively, for MN-like cells derived from tonsil mesenchymal stem cells (T-MSCs). Also, other studies show 35–46% and 70–90% positive cells for HB9 and ISL-1, respectively, in MN-like cells derived from induced pluripotent stem cells (iPSCs)^85–87^.

Furthermore, the neurite length of MN-like cells in the 2-Col/PA-CH/Mic EpoB group increased remarkably to 146 µm, which is more than three times longer compared to the other two groups without Epo B microspheres (2-Col/PA-CH, and collagen groups). Recently, it was reported that acellularized spinal cord scaffolds incorporating bpV(pic)/PLGA microspheres promote axonal regeneration by about 45 µm^88^.

Another study indicated that adding 3 nM of paclitaxel as an MSA can enhance neurite length of mature rat retinal ganglion cells (RGCs) by 40 µm while using 10 nM paclitaxel significantly decreases neurite outgrowth^89^. This shows that paclitaxel has less of an effect on neurite outgrowth than EpoB. Additionally, compared with paclitaxel, EpoB has a smaller molecular size, higher solubility, and the capability to pass the blood–brain barrier (BBB)^90,91^. It is worth noting that EpoB can be effective for axon elongation and MN-differentiation, despite the presence of the neurite outgrowth inhibitor A, which is known to be an axonal inhibitor in SCI^23,26^.

One proposed method of increasing neurite outgrowth in recent years is electrical stimulation^92–94^. A study reported that the neurite length of PC12 cells via gold-nanoparticle after electrical stimulation (ES) was 120 µm^95^. Another study reported, the final neurite length of the primary prefrontal cortex (PFC) in collagen 3D cultures with ES to be ~ 110 µm, compared to the control wild-type where only ~ 10 µm of elongation was observed^96^. Thus, our results indicate that the fabricated 2-Col/PA-CH/Mic EpoB hydrogel achieved a higher neurite elongation than the mentioned studies.

While understanding the exact mechanism of EpoB and adapting it to the complex mechanism of SCI yet need to achieve a clear vision, today its role in stem cell differentiation and SCI has garnered attention, and our study demonstrated the enhanced MN differentiation efficiency of hEnSCs and neurite growth by 3D cross-linked collagen hydrogel containing EpoB-loaded microspheres which can be used as a combinatorial approach for MN-diseases modeling and a future aid in SCI repair.

## Conclusion

To our knowledge, this is the first time that ultra-low doses of EpoB loaded microspheres have been synthesized for the purpose of MN differentiation from hEnSCs. Our study demonstrates that incorporation of EpoB microspheres with collagen hydrogel can significantly increase the MN differentiation efficiency of hEnSCs by up-regulating *ISL-1* and *HB9*, known as mature motor neuron key markers. Moreover, our results showed that the addition of EpoB microspheres significantly enhances neurite growth during MN differentiation. Utilizing proanthocyanidin and calcium hydroxide as cross-linkers significantly reduces the degradation degree of the 3D collagen hydrogel and provides an appropriate stable ECM for supporting cell attachment, proliferation, and differentiation. Our findings emphasize the active role that 3D cross-linked collagen hydrogels containing EpoB-loaded microspheres for nerve tissue engineering and the potential of using combinatorial approaches to overcome the limitations of individual methods for SCI repair.

## Data Availability

The datasets used and/or analyzed during the current study are available on reasonable request**.**
